# Aberrant Expression of Long Non-coding RNAs in Exosomes in Follicle Fluid From PCOS Patients

**DOI:** 10.3389/fgene.2020.608178

**Published:** 2021-02-17

**Authors:** Liping Wang, Hairui Fan, Yinggang Zou, Qiuyue Yuan, Xuming Hu, Xujing Chen, Chunhui Zhu, Xiaomei Zhang, Hengmi Cui

**Affiliations:** ^1^Department of Biobank Clinical Medical College, Yangzhou University, Yangzhou, China; ^2^Institute of Epigenetics and Epigenomics, College of Animal Science and Technology, Yangzhou University, Yangzhou, China; ^3^College of Animal Science and Technology, Yangzhou University, Yangzhou, China; ^4^Department of Obstetrics and Gynecology, The Second Hospital of Jilin University, Changchun, China; ^5^College of Pharmacy, Dalian Medical University, Dalian, China

**Keywords:** polycystic ovary syndrome, follicle fluid, exosomes, long non-coding RNAs, bioinformatics

## Abstract

Polycystic ovary syndrome (PCOS) is a common reproductive endocrine disease characterized by persistent anovulation and hyperandrogenism, affecting approximately 8–10% of women of childbearing age and occupying an important position in the etiology of infertility. There is increasing evidence that long non-coding RNAs (lncRNAs) are involved in the development of PCOS, but the potential regulatory mechanism is still unclear. This study performed high-throughput lncRNA sequencing of follicular fluid exosomes in non-PCOS infertility patients and PCOS infertility patients. The sequencing results led to the identification of 1,253 upregulated and 613 downregulated lncRNAs from a total of 1,866 detected candidates. There was no significant difference between the PCOS patients and non-PCOS patients in body mass index (BMI) or the fasting blood glucose (FBG) level. However, luteinizing hormone (LH), estradiol (E2), testosterone (T), serum prolactin (PRL), and anti-Mullerian hormone (AMH) levels were clearly upregulated in PCOS patients compared to those in non-PCOS patients. There was also an increase in LH/FSH (>2) in the PCOS patients. Functional analysis showed pathways related to endocytosis, the Hippo, the MAPK, and HTLV-1 infection. These results suggest that lncRNAs may play an important role in the pathogenesis of PCOS and may be potential targets for the diagnosis and treatment of PCOS.

## Introduction

Polycystic ovary syndrome (PCOS) is a common reproductive endocrine disease in women of childbearing age, accounting for 70–80% of patients with ovulatory disorders, and plays an important role in the etiology of infertility (Ehrmann, 2005). The pathological changes of PCOS include abnormal follicular granulosa cell hyperproliferation and apoptosis, hyperandrogenism, and ovulation disorders. Meanwhile, PCOS is characterized by its phenotypical heterogeneity, in which patients often exhibit diverse symptoms such as menstrual irregularities, amenorrhea, abnormal hair growth, or hair loss as well as obesity (Jaliseh et al., 2017; Blagojević et al., 2018). PCOS symptoms in patients coupled with those of other metabolic disorders and infertility could lead to disturbances of social and family life. Therefore, it is of the utmost importance to reveal the underlying mechanisms that regulate PCOS. Previous scientific data have urged the combination of genetic and environmental factors to explore molecular mechanisms in PCOS patients (Chen et al., 2011; Rutkowska and Diamanti-Kandarakis, 2016). Although the Rotterdam standard applies to clinical diagnosis, the clinical manifestations of patients are highly heterogeneous due to differences in regions and races. Early diagnosis and treatment are desirable for PCOS patients. In light of the above considerations, we argue that it is important to screen and identify specific markers associated with the pathogenesis of PCOS.

Follicular fluid is the direct internal environment of oocytes during follicular development, and it directly affects oocyte quality and clinical pregnancy outcomes in assisted reproductive patients. It also plays an important role in reproductive function (Rodrigues et al., 2010). Studies have shown that a variety of lipids, proteins, cytokines, and other molecules found in follicular fluid are closely related to follicular development, embryo quality, *in vitro* fertilization outcomes, and female reproductive system diseases (Cavallo et al., 2017; Jaliseh et al., 2017). For instance, a genome-wide deep sequencing study by Sang et al. (2013) revealed that there are numerous miRNAs present in the follicular fluid, some of which play a role in steroidogenesis. Furthermore, Naji et al. (2017) screened differentially expressed miRNAs in human follicular fluid and found that they were correlated significantly with hormone levels in PCOS patients. They also had good predictive value for determining the pathogenesis of PCOS. In the early stage of this project, long non-coding RNAs (lncRNAs) in the follicular fluid of PCOS patients of reproductive age were collected, and high-throughput lncRNA sequencing analysis was conducted. The results were compared with the sequences of lncRNAs from non-PCOS patients. The sequencing results led to the identification of 1,253 upregulated and 613 downregulated lncRNAs among 1,866 detected candidates. This study suggests that the information carried in follicular fluid is an important entry point for the study of PCOS-induced follicular dysplasia and ovulation disorders and may be an important research subject for screening specific PCOS-associated markers.

Exosomes are globular or cup-shaped vesicles with a double-layered membrane and have diameters of approximately 30–100 nm. Studies have shown that they are widely found in blood, lotions, placenta, and amniotic fluid. They can also be isolated from peritoneal effusions and follicular fluids (Vlassov et al., 2012; Wang et al., 2019a). Exosomes can release biologically active substances such as mRNA, miRNA, and lncRNA and can participate in the development of immune and intercellular communication, cell proliferation, cell migration, cell differentiation, and metabolic diseases (Li et al., 2018). Da Silveira et al. (2012) first discovered that porcine follicle-derived exosomes contain miRNA and protein and found that ovarian granulosa cells could absorb exosomes extracted from follicular fluid. The study also confirmed that miR-181A, miR-375, and other miRNAs displayed decreased expression with age in mares, thus demonstrating that the miRNAs carried by exosomes are age-related. Subsequent extraction of a large number of miRNAs from bovine follicular fluid exosomes revealed the high expression of miR-654-5p and miR-640 during follicle growth, and the high expression of miR-373 in mature follicular fluid exosomes indicates that exosomes extracted from follicular fluid contribute to the growth and development of follicles (Sang et al., 2013; Hung et al., 2015). It is of great significance to detect the specific RNA expression in exosomes from follicular fluid to assist in the early diagnosis of PCOS patients.

Increasing evidence indicates that epigenetic regulation is closely related to the development of reproductive system diseases. lncRNA is an RNA molecule with no protein-encoding transcript, and it has more than 200 nucleotides and is a key regulator of many biological processes, such as genomic imprinting, DNA metabolism, X-chromosome inactivation, transcriptional activation, and chromatin modification (Subramanian et al., 2013; Lee et al., 2016). Studies have shown that specific lncRNAs play an important role in the pathogenesis of cervical cancer, endometrial cancer, and ovarian cancer (Sun et al., 2017; Wu et al., 2019). According to reports, the expression level of the lncRNA ILF3-AS1 is correlated with the stage and differentiation of cervical cancer (Wu et al., 2019). The lncRNA LRRC8C-DT plays a key role in the progression of endometrial cancer (Chen et al., 2016; Sun et al., 2017). Therefore, we speculated that lncRNAs may be important in follicular development. However, no study has investigated whether lncRNAs are also implicated in the pathogenesis of PCOS. Therefore, we believe that the in-depth study of lncRNAs and their regulatory mechanisms during the development of PCOS will greatly promote the diagnosis and treatment of PCOS.

In this study, bioinformatics analysis was used to compare the difference in lncRNA expression in follicular fluid exosomes between non-PCOS infertility patients and PCOS infertility patients. Our results showed that endocytosis, the Hippo signaling pathway, the MAPK signaling pathway, and HTLV-1 infection could be targeted by differential lncRNAs and contribute to PCOS pathogenesis. This study provides useful guidance for further in-depth research on various genes and pathways that contribute to the development and progression of PCOS.

## Materials and Methods

### Group of Study Subjects

We collected the follicular fluids generated during IVF treatment cycles in Northern Jiangsu People’s Hospital from 1st January to 30th May 2017. All patients were younger than 34 years old. The inclusion criteria for the PCOS group were the revised 2003 criteria (two out of three): rare ovulation or anovulation; clinical and/or biochemical signs of hyperandrogenism; and ultrasound examination of the ovary on the 3rd to 5th day of a menstrual cycle or after bleeding following progesterone withdrawal showing the following ovarian polycystic changes: in the unilateral or bilateral ovaries, a number of follicles with a diameter of 2–9 mm ≥12 and/or an ovarian volume ≥10 ml. In addition, exclusion of other diseases that cause androgen excess and/or low gonadotropin-induced anovulation and premature ovarian failure was made. The inclusion criteria of the control group were normal ovarian function, a regular menstrual cycle (26–32 days), normal basal reproductive hormone levels, and a normal number of basal ovarian follicles (unilateral AFC = 6–10). All the selected patients were diagnosed with either tubal factor infertility or male factor infertility. Common exclusion criteria: women older than 34 years, ovarian cysts, ovarian tumors, history of ovarian surgery, history of ovarian radiotherapy, and chemotherapy, endometriosis, hyperthyroidism/hypothyroidism, hyperprolactinemia, endocrine diseases, and chromosomal abnormalities. The study was approved by the Clinical Research Ethics Committee of Northern Jiangsu People’s Hospital, and the human tissues were obtained with informed consent.

### The Collection and Treatment of Follicular Fluid Exosomes

In this study, six infertility patients had their follicular fluids collected during treatment with assisted reproductive technology. After standing for 30 min at room temperature, the fluids were centrifuged at 3,000 r/min for 10 min at 4°C. Then, the supernatant was transferred into a clean Amicon Ultra 30 K Centrifugal Device (Millipore, Bedford, MA) and centrifuged at 10,000*g* at 4°C for 30 min. In addition, the supernatant was mixed with 0.2 vol of total exosome isolation reagent (Invitrogen, Carlsbad, CA). Then, the resulting mixture was incubated at room temperature for 30 min before being centrifuged at 10,000*g* at 4°C for 30 min. Finally, the supernatant was discarded, and the pellet was resuspended in phosphate-buffered saline in a 1.5 ml microcentrifuge tube to a final volume equivalent to one fourth of that of the concentrated exosome sample solution. The obtained exosome samples were immediately used for the next experiment.

### High-Throughput lncRNA Sequencing

The RNA of follicular fluid exosomes was extracted (batch number: 151012397, Qiagen, United States), and the total RNA purity and concentration were measured. After passing the total RNA quality test, the ribosomal RNA (batch number: 7E010H6, Nanjing Vazyme Co., Ltd.) was removed, and the RNA was fragmented (batch number: 0006358486, Agilent, United States). The cDNA was then synthesized using the fragmented dsRNA as a template, and the final sequencing library was obtained by PCR amplification. After quality inspection, the library was sequenced using an Illumina HiSeq 2500 (Illumina, United States), and the sequencing read length was 2 × 150 bp (double-ended) (PEl50). The size of the clean dataset from each sample was at least 10 G. HISAT + StringTie + Ballgown analysis software (Johns Hopkins Biocomputing Center, United States) enables efficient alignment of RNA-Seq reads, especially across multiple exons. The complete lncRNA sequencing datasets are available on the NCBI GEO database GEO: GSE159466. The sample included in this study was sequenced three times, and all test procedures were performed in strict accordance with the kit instructions and instrument specifications.

### Real-Time Quantitative Polymerase Chain Reaction (RT-qPCR)

According to the manufacturer’s instructions, total RNA from exosomes in follicular fluid was extracted by using TRIzol reagent (Invitrogen, Carlsbad, CA, United States). Subsequently, the extracted total RNA was reverse-transcribed into cDNA according to the instructions of the Reverse Transcription Kit (TaKaRa, Dalian, China) for RT-qPCR. The primer sequences are shown in Table 1. The thermocycling conditions were as follows: 30 s at 95°C and then 40 cycles of 5 s at 95°C and 35 s at 60°C.

**TABLE 1:** Information of PCOS patients and non-PCOS donors for RNA sequencing.

Project	Non-PCOS	PCOS
	3	3
Age (years)	27 ± 1.0	25.3 ± 1.5
BMI (Kg/m^2^)	20.4 ± 3.8	23.0 ± 3.1
LH (IU/L)	5.9 ± 3.6	11.1 ± 0.8
FSH (IU/L)	6.6 ± 1.7	5.9 ± 0.8
E2 (pmol/L)	122.7 ± 13.2	155.3 ± 16.6
T (nmol/L)	1.0 ± 0.4	2.1 ± 0.9
FBG (mmol/L)	5.2 ± 0.1	5.0 ± 0.2
Infertility (years)	3.7 ± 0.6	2.3 ± 1.2
PRL (ng/mL)	23.1 ± 5.5	37.0 ± 6.5
AMH (ng/mL)	9.2 ± 0.6	11.7 ± 3.2
Number of follicles	13.0 ± 0.6	23.7 ± 0.3

### Functional Enrichment Analysis

To explore the functions and pathways related to the differentially expressed lncRNAs, we performed gene ontology (GO)^1^ term and Kyoto Encyclopedia of Genes and Genomes (KEGG)^2^ pathway analyses by using the DAVID bioinformatics tool (version 6.8)^3^. The threshold was set as a *P* < 0.05.

### Statistical Analysis

Statistical Product and Service Solutions (SPSS) 20.0 was used for all statistical analyses. Differences in the lncRNA expression profile between the PCOS group and the non-PCOS group were analyzed using Student’s *t*-test, and all RT-qPCR amplifications were performed in triplicate. *p* < 0.05 was considered statistically significant.

## Results

### Comparison of the General Clinical Data Between the Two Groups of Patients

To ensure the reliability of the study results, the selected samples of this study were strictly screened according to the Rotterdam criteria (2003). There were two groups (non-PCOS patients = 3, PCOS patients = 3) of patients, and their follicular fluid exosome samples were used as the research objects. All enrolled participants were diagnosed with primary infertility and received the same ovulation induction treatment program; they were aged between 24 and 28 years with a duration of infertility between 1 and 4 years. The results showed that there was no significant difference between the PCOS patients and the non-PCOS patients in body mass index (BMI) or the fasting blood glucose (FBG) level. The LH and T levels were significantly different between the two groups. In addition, the luteinizing hormone (LH), estradiol (E_2_), testosterone (T), serum prolactin (PRL), and anti-Mullerian hormone (AMH) levels were clearly upregulated in the PCOS patients, and there were patients with a LH/FSH > 2 in the PCOS group. The general clinical data of the two groups of patients are displayed in Table 1.

### lncRNA Screening and Identification

**FIGURE 1:**
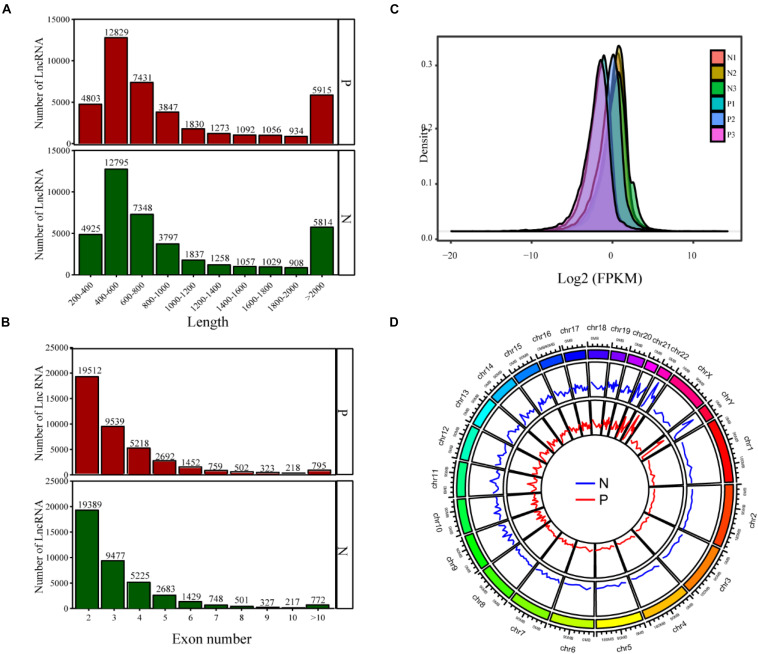
The lncRNA sequencing results show the distribution of lncRNAs based on length **(A)**, exon number **(B)**, FPKM value **(C)**, and chromosomal location **(D)**. P, PCOS patients; N, non-PCOS patients.

The lncRNAs were divided into two types: intergenic lncRNAs and intragenic lncRNAs. The identified lncRNAs displayed a length distribution between 900 and 13,000 bp (Figure 1A), and the distribution of all lncRNAs according to the number of exons is shown in Figure 1B, indicating that the majority of the lncRNAs encompassed no more than two exons. lncRNAs derived from non-PCOS patients showed higher density values than those detected in infertile PCOS individuals (Figure 1C). Overall, the lncRNAs were shown to be abundantly present in all chromosomes (Figure 1D). Notably, the lncRNAs detected in the PCOS patients and controls exhibited highly similar chromosomal distribution patterns (Figure 1D).

### Differential Expression Analysis of Exosomal lncRNAs in the Two Groups

**FIGURE 2:**
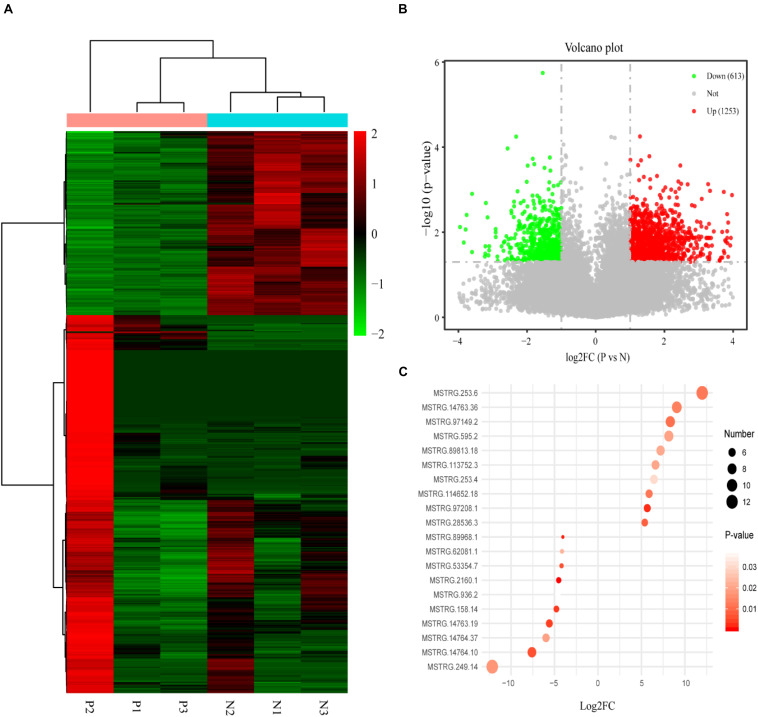
**(A)** The heat map shows the hierarchical clustering analysis of lncRNAs detected in individual PCOS patients (P1–3) and non-PCOS patients (N1–3). **(B)** The volcano map shows the distribution of the differentially expressed lncRNAs according to their *P* values and fold changes. Candidates with a *P* < 0.05 and a | log2 fold change| ≥ 1 are considered differentially expressed. **(C)** The RPM values of the top 10 most up- and downregulated lncRNAs.

**FIGURE 3:**
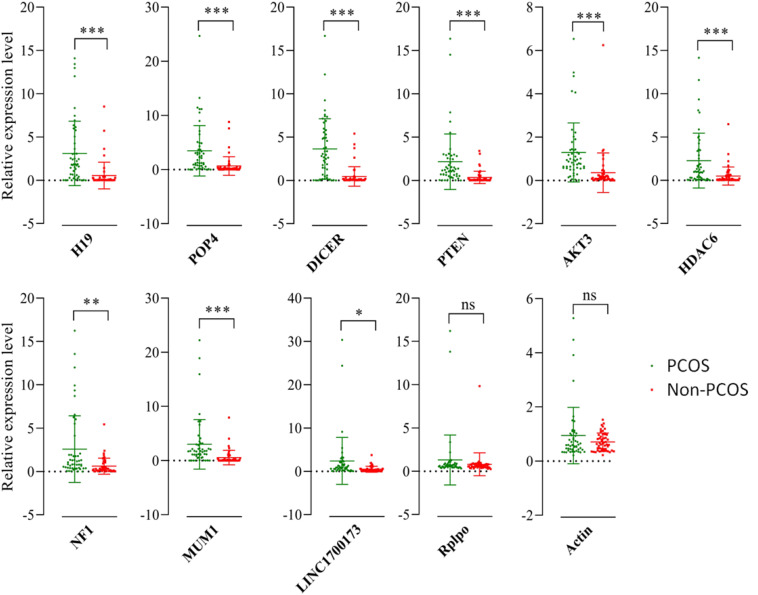
Validation of selected lncRNAs by qRT-PCR. Red and blue dots represent the data from PCOS patients and non-PCOS patients, respectively. ^∗^*P* < 0.05; ^∗∗^*P* < 0.01; ^∗∗∗^*P* < 0.001.

There were 1,866 differentially expressed lncRNAs in follicular fluid exosomes in the PCOS group and non-PCOS group, among which the number of upregulated lncRNAs was 1,253 and the number of downregulated lncRNAs was 613 (Figure 2). To further validate our lncRNA profiling results and determine the biological function of lncRNAs in the development of PCOS, nine lncRNAs (lncRNA-H19, lncRNA-POP4, lncRNA-DICER1, lncRNA-PTEN, lncRNA-AKT3, lncRNA-HDAC6, lncRNA-NF1, lncRNAMUM1, and lncRNA-LINC00173) were preliminarily screened by RT-qPCR according to a lncRNA expression differential multiple value >10 and a *P* < 0.0001 in follicular fluid exosomes from 25 randomly chosen PCOS patients and 25 non-PCOS patients. According to the RT-qPCR results, all lncRNAs were more highly expressed in PCOS follicular fluid exosome samples than in non-PCOS follicular fluid exosome samples (Figure 3). Therefore, the expression trends of all the lncRNAs determined by RT-qPCR were identical to those obtained from RNA sequencing (Figure 3).

### Functional Annotation and Identification of the Differentially Expressed lncRNAs

**FIGURE 4:**
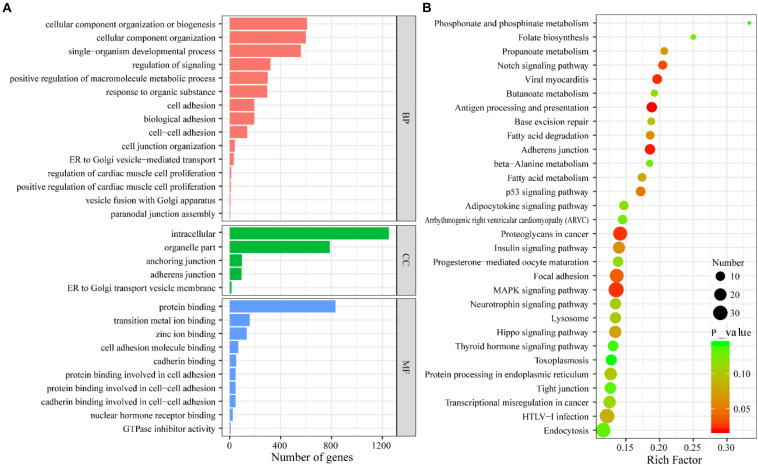
**(A)** GO enrichment analysis of differentially expressed lncRNAs. **(B)** KEGG pathway analysis of differentially expressed lncRNAs.

GO enrichment and KEGG pathway analyses were carried out to gain insight into the biological characteristics of the lncRNAs. Then, pathways related to endocytosis, the Hippo signaling pathway, the MAPK signaling pathway, and pathways associated with HTLV-1 infection were examined. Meanwhile, there were five genes, CRK, CDC25B, AKT2, ELK4, and TGFBR1, that showed a possible connection to the MAPK signaling pathway with high enrichment scores. This suggests that these pathways could contribute to PCOS pathogenesis. The complete information is shown in Figure 4.

### Construction of the lncRNA–miRNA–mRNA Network

**FIGURE 5:**
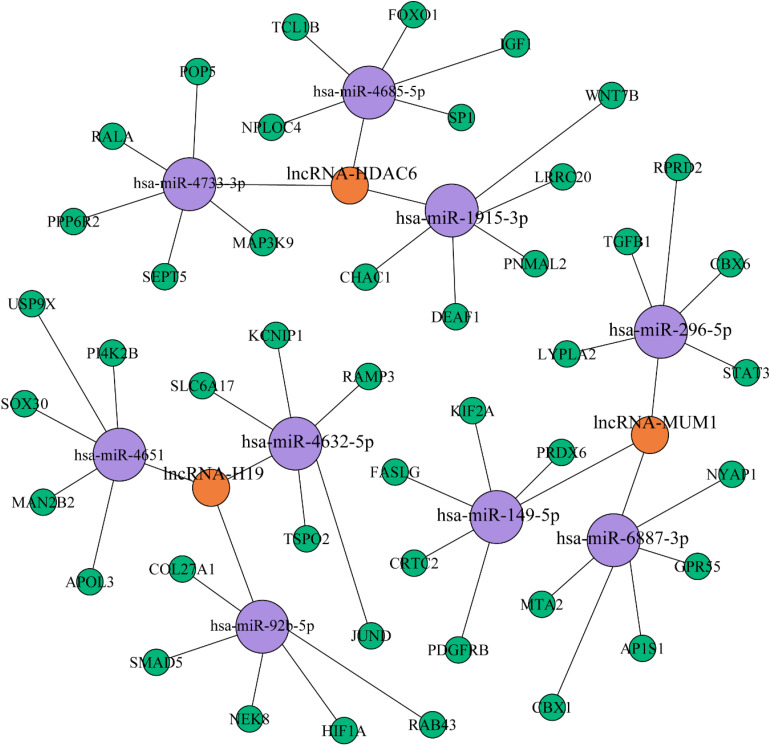
lncRNA–miRNA interaction network based on the validated lncRNAs. The purple and blue circles represent lncRNA and miRNA targets, respectively. The interaction between a lncRNA and one of its potential miRNA targets is denoted by a red line. The thickness of the line indicates the number of potential binding sites in the miRNA target, whereas the intensity of its color denotes the strength of the interaction.

Subsequently, Gephi was used to construct a lncRNA–miRNA interaction network based on the validated lncRNA candidates. To further investigate the interconnections between the differentially expressed lncRNAs and miRNAs involved in PCOS, Pearson correlation coefficients were applied using the following two criteria. First, the absolute value of the correlation coefficient was greater than 0.99, and second, the *P*-value was less than 0.001; these were the thresholds used to determine the correlation between the lncRNAs and miRNAs and to draw a network diagram. The coexpression network suggests that one lncRNA can interact with dozens of coding genes, and conversely, one coding gene can also be associated with multiple lncRNAs; therefore, we speculate that there is a correlation between the expression profiles of miRNAs and lncRNAs involved in PCOS. As a result, the network contained five validated lncRNAs (Figure 5). lncRNA-H19 represented the largest node and was predicted to have the potential to interact with 15 target miRNAs.

The thickness and relative intensity of each connection represented the number of potential binding sites and the release of free energy resulting from the lncRNA–miRNA interaction, respectively. We aimed to identify the key genes and miRNAs that affect the differential expression of lncRNAs involved in the occurrence of PCOS by constructing the lncRNA–miRNA–mRNA network. miR-4651 may be involved in inflammation via leukocyte transendothelial migration by regulating its target gene (Wang et al., 2019b). Furthermore, upregulation of miR-1915 inhibits the proliferation, invasion, and migration of *Helicobacter pylori*-infected gastric cancer cells by targeting RAGE (Xu et al., 2019). These findings may indicate new research directions and constitute a breakthrough in the study of the pathogenesis of PCOS.

## Discussion

PCOS is one of the most common heterogeneous reproductive and metabolic diseases, affecting approximately 5–10% of women of childbearing age worldwide (Ehrmann, 2005). The clinical manifestations of the syndrome are highly variable, including hyperandrogenism, irregular menstruation, and polycystic ovary morphology. Women with PCOS are often infertile, obese, and hirsute and have insulin resistance (IR) and dyslipidemia. In addition, the risks of endometrial cancer, type 2 diabetes mellitus, and cardiovascular disease are increased. However, the exact pathophysiological mechanism of PCOS remains unclear. At present, clinical treatment is mainly based on conservative treatment involving the adjustment of the menstrual cycle, the promotion of ovulation, the improvement of living habits, and the prevention of long-term complications. lncRNAs have been shown to be key regulatory factors in many biological processes, such as genomic imprinting regulation, DNA metabolism, X chromosome inactivation, transcriptional activation, and chromatin modification. Studies have shown that differentially expressed lncRNAs are involved in a variety of human diseases, such as cancer and diseases of the cardiovascular, neurological, and reproductive systems, but the specific mechanism is still unclear. The exploration of dysregulated lncRNAs and their functions in related diseases has attracted increasing attention and may be helpful in the discovery of new diagnostic markers and therapeutic targets for PCOS.

The exosome is a tiny membrane bubble that can be secreted by most cells. It has a lipid bilayer membrane structure that contains cell-specific proteins, lipids, and nucleic acids. It acts as a signaling molecule that can migrate to other cells to change their function (De la Torre Gomez et al., 2018). In this study, the lncRNA expression profile of the exosomes in follicle fluids obtained from PCOS patients and non-PCOS patients was selected to comprehensively analyze the potential role of lncRNAs in PCOS. There were 1,866 differentially expressed lncRNAs in follicular fluid exosomes from the PCOS group and non-PCOS group, among which the number of upregulated lncRNAs was 1,253 and the number of downregulated lncRNAs was 613 according to RNA sequencing. Furthermore, the results for nine lncRNAs (lncRNA-LINC00173, lncRNA-H19, lncRNA-HDAC6, lncRNA-POP4, lncRNA-PTEN, lncRNAAKT3, lncRNA-DICER1, lncRNA-NF1, and lncRNA-MUM1) with significant changes were verified by RT-qPCR, and all these lncRNAs were much more highly expressed in PCOS follicular fluid exosome samples than in non-PCOS follicular fluid exosome samples. In addition, the expression trends were identical to those obtained from RNA sequencing. Therefore, bioinformatics analyses were carried out to gain insight into the biological characteristics of lncRNAs. Then, the pathways related to endocytosis, Hippo signaling, MAPK signaling, and HTLV-1 infection were examined. By constructing the lncRNA–miRNA–mRNA network, we demonstrated that miR-146a-5p, miR-4632, and miR-92-5p were closely related to the occurrence of PCOS. The parietal granulosa cells and cumulus cells of human primary follicles were isolated from healthy women during *in vitro* fertilization (IVF), and the miRNA expression profile sequencing was conducted by Andrei et al. Their results showed that there were significant differences in the expression of miR-146a-5p and miR-149-5p between cumulus and mural granulosa cells from human preovulatory follicles (Andrei et al., 2018). Other studies have suggested that miR-4632 plays an important role in regulating HPASMC proliferation and apoptosis by suppressing cJUN, thus providing a novel therapeutic miRNA candidate for the treatment of pulmonary vascular remodeling diseases (Qian et al., 2017). Moreover, a luciferase activity assay revealed that miR-92b can directly bind to lncRNA-PWRN2 and play vital roles in oocyte maturation in PCOS (Huang et al., 2018). Our findings provide new information and clarify the pathogenesis of PCOS.

## Conclusion

In summary, our current study analyzed the differentially expressed lncRNAs in the follicular fluid of PCOS patients through RNA sequencing and bioinformatics. We verified their close correlation with endocytosis, the Hippo signaling pathway, the MAPK signaling pathway, and HTLV-1 infection. These results could provide useful guidance for further in-depth research on the various genes and pathways that contribute to the development and progression of PCOS.

## Data Availability Statement

The RNA sequencing data has been deposited to GEO (accession: GSE159466).

## Ethics Statement

The studies involving human participants were reviewed and approved by Northern Jiangsu People’s Hospital ethics committee. The patients/participants provided their written informed consent to participate in this study. The animal study was reviewed and approved by Clinical Research Ethics Committee of Northern Jiangsu People’s Hospital, and the human tissues were obtained with informed consent.

## Author Contributions

HC and XZ conceived the idea. LW, HC, and XZ wrote the manuscript. HF and YZ edited and revised the manuscript. QY and XH analyzed the data. XC and CZ collected the data. All authors listed have made a substantial, direct and intellectual contribution to the work, and approved it for publication.

## Conflict of Interest

The authors declare that the research was conducted in the absence of any commercial or financial relationships that could be construed as a potential conflict of interest.

## Footnotes

1 http://geneontology.org/

2 http://www.genome.jp/kegg/

3 https://david.nciferf.gov/home.jsp
