# Supplementary material for: Aberrant Expression of Long Non-coding RNAs in Exosomes in Follicle Fluid From PCOS Patients
Source: Front Genet. 2021 Feb 17;11:608178. doi: 10.3389/fgene.2020.608178 (PMC7925891; doi:10.3389/fgene.2020.608178)
Supplement: Supplementary file 1 [file Table_1.DOCX]

**Tab.S1 Information of PCOS patients and Non-PCOS donors for RT-qPCR**

| **Project** | **PCOS** | **Non-PCOS** |
| --- | --- | --- |
|  | 3 | 3 |
| **Age（years）** | 26.5±1.6 | 28.9±3.2 |
| **BMI(Kg/m^2^)** | 24.9±3.7 | 21.5±2.2 |
| **LH(IU/L)** | 10.9±1.6 | 4.4±1.2 |
| **FSH(IU/L)** | 5.7±1.2 | 6.6±1.5 |
| **E2(pmol/L)** | 159.7±18.5 | 125.2±11.7 |
| **T(nmol/L)** | 2.3±0.7 | 1.1±0.4 |
| **FBG(mmol/L)** | 5.6±0.6 | 4.8±0.5 |
| **Infertility(years)** | 3.0±1.4 | 2.9±1.1 |
| **PRL(ng/ml)** | 38.6±6.9 | 24.7±5.3 |
| **AMH(ng/ml)** | 12.3±2.8 | 9.5±0.7 |
| **Number of follicles** | 21.8±1.5 | 8.4±2.2 |
